# Distinct phenotypic subpopulations of circulating CD4^+^CXCR5^+^ follicular helper T cells in children with active IgA vasculitis

**DOI:** 10.1186/s12865-016-0176-6

**Published:** 2016-10-21

**Authors:** Deying Liu, Jinxiang Liu, Jinghua Wang, Congcong Liu, Sirui Yang, Yanfang Jiang

**Affiliations:** 1 Department of Pediatric Rheumatology and Allergy, The First Affiliated Bethune Hospital of Jilin University, Changchun, 130021 China; 2 Genetic Diagnosis Center, The First Hospital of Jilin University, Changchun, 130021 China; 3 Key Laboratory of Zoonoses Research, Ministry of Education, The First Hospital of Jilin University, Changchun, 130021 China; 4 Jiangsu Co-innovation Center for Prevention and Control of Important Animal Infectious Diseases and Zoonoses, Yangzhou, 225009 China

**Keywords:** Follicular helper T cells, IgA vasculitis, Interleukin 21, Symptoms, Remission, Glucocorticoid

## Abstract

**Background:**

Circulating follicular helper T (Tfh) cells are a heterogeneous population of CD4^+^ helper T cells that promotes pathogenic immune responses in autoimmune diseases. In this study, we examined the status of different subpopulations of Tfh cells in peripheral circulation and their associations with various clinical characteristics of IgA vasculitis (IgAV).

**Methods:**

According to the phenotypic expressions of different molecules, focus was given on six subpopulations of Tfh cells: CD4^+^CXCR5^+^, CD4^+^CXCR5^+^ICOS^+^, CD4^+^CXCR5^+^ICOS^+^PD-1^+^, CD4^+^CXCR5^+^ICOS^high^PD-1^high^, CD4^+^CXCR5^+^ICOS^−^PD-1^+^, and CXCR5^+^CD45RA^−^IL-21^+^. The frequencies of these six subpopulations and the circulating level of Tfh-related cytokine interleukin 21 (IL-21) were measured from 27 patients with IgAV and 15 healthy controls (HC) by flow cytometry and flow cytometric bead array, respectively.

**Results:**

Significantly higher frequencies of CD4^+^CXCR5^+^, CD4^+^CXCR5^+^ICOS^+^, CD4^+^CXCR5^+^ICOS^+^PD-1^+^, CD4^+^CXCR5^+^ICOS^high^PD-1^high^ and CXCR5^+^CD45RA^−^IL-21^+^ Tfh cells, as well as higher levels of plasma IL-21, were detected in IgAV patients compared to HC. The level of each Tfh subpopulation varied by the presenting symptoms of IgAV, but did not differ between patients treated or not treated with glucocorticoids. When the disease entered the remission stage following treatment, circulating levels of CD4^+^CXCR5^+^, CD4^+^CXCR5^+^ICOS^+^, CD4^+^CXCR5^+^ICOS^+^PD-1^+^, CD4^+^CXCR5^+^ICOS^high^PD-1^high^ and CXCR5^+^CD45RA^−^IL-21^+^ Tfh cells, as well as plasma IL-21 levels were reduced. Among the six subpopulations of Tfh cells, both CD4^+^CXCR5^+^ICOS^+^ and CXCR5^+^CD45RA^−^IL-21^+^ significantly and positively correlated with serum IgA and plasma IL-21 levels, but only CXCR5^+^CD45RA^−^IL-21^+^ significantly and negatively correlated with the serum C4 level.

**Conclusions:**

Tfh cells may differentially contribute to the development of IgAV or predict disease progression. These findings provide novel insights in the pathogenesis of IgAV and may benefit treatment development targeting organ-specific presenting symptoms of IgAV.

## Background

Immunoglobulin A vasculitis (IgAV), also known as Henoch-Schönlein purpura, is an autoimmune disease caused by the deposition of IgA-dominant immune complexes in small vessels [1, 2]. It is the most common cutaneous vasculitis in children, and its annual incidence is 13–20 per 100,000 children under 17 years old [3]. IgAV usually develops following the upper respiratory infection of viruses, bacteria, parasites, or others; with the common ones being group A streptococci, Mycoplasma, Epstein-Barr virus, Varicella virus and others [4]. The clinical features of IgAV are characterized by a tetrad of non-thrombocytopenic palpable purpura (most commonly located on the lower extremities and buttocks, skin involvement), arthralgia/arthritis (joint involvement), bowel angina (gastrointestinal involvement), and hematuria/proteinuria (renal involvement) [5]. Therapy for IgAV is mostly supportive and symptomatic, because the disease is usually benign and self-limited. For patients with severe active symptoms in one or multiple organs, glucocorticoids (GC) are administered to improve the treatment effect. Complications are rare. However, complications resulting from blood vessel lesions in different organ systems could sometimes be severe, of which, renal involvement is the most serious complication and the principle cause of mortality in IgAV patients [6–8].

Although the pathogenesis of IgAV is not completely understood, it is clear that both the aberrant deposition of glycosylated IgA in small vascular walls and the subsequent activation of an alternate complement pathway play a central role in IgAV development [9]. Multiple immune cell types including CD4^+^ helper T (Th) cells, B cells and natural killer (NK) cells are implicated in the pathogenesis of IgAV [10]. Furthermore, Th1/Th2 imbalance, the hyperactivity of Th2 cells and the decline in the ratio of CD4^+^/CD8^+^ cells increase the synthesis and release of immunoglobulins in IgAV patients. The increased frequency of peripheral Th17 cells and serum IL-17 levels were also observed in childhood IgAV [11].

Follicular helper T (Tfh) cells are a subset of CD4^+^ Th cells that are specialized in helping B cell responses to produce antigen-specific antibodies such as IgA, IgE, IgG and IgM in autoimmune diseases, infectious diseases, and tumors [12–14]. Although no unique markers have been reported for Tfh cells, they could be identified through a combination of markers closely related to their functions including chemokine receptor CXCR5, programmed death-1 (PD-1), inducible costimulator (ICOS), SLAM adapter protein (SAP), B and T lymphocyte attenuator (BTLA), CD40 ligand (CD40L) and cytokine interleukin 21 (IL-21). Originally identified in germinal centers of secondary lymphoid organs and essential for germinal center formation, B-cell affinity maturation, class switch recombination, and the generation of plasma and memory B cells [15–17], Tfh counterparts were recently detected in tonsils [18] and blood circulations [19, 20]. The expansion of circulating Tfh cells has been reported in various autoimmune diseases [19, 21], suggesting their pathogenic significance. Xie et al. revealed that the frequency of circulating CD4^+^CXCR5^+^ICOS^+^ Tfh cells in children with active IgAV was significantly higher than in healthy children [22]. Wang et al. also reported that the upregulation of circulating Tfh cells and downregulation of circulating follicular regulatory T (Tfr) cells may contribute to the pathogenesis of IgAV in children [23]. At present, many questions remain to be addressed in regard to the expansion of circulating Tfh cells in autoimmune diseases. When Tfh cells are characterized by common markers, are we obtaining a homogenous or heterogeneous population of Tfh cells? If Tfh cells are composed of heterogeneous subpopulations, as suggested by other studies [20], is each subpopulation functionally equivalent in the pathogenesis of a specific autoimmune disease? The answers to these questions would improve our understanding not only on Tfh cells and their functions, but also on their specific contribution to autoimmune diseases, which would facilitate therapeutic development.

In order to address these questions, in this study, we focused on six phenotypic subpopulations of circulating Tfh cells as defined by the expressions of distinct molecules, examined their associations with IgAV, specifically the different dominant symptoms presented in IgAV, and explored the correlations between these Tfh subpopulations and key IgAV clinical parameters.

## Methods

### Patients

The experimental protocols were established following the Declaration of Helsinki and approved by the Human Ethics Committee of Jilin University (Changchun, China). Written informed consent was obtained from all participants. A total of 27 patients with newly diagnosed active IgAV admitted to the inpatient care of the Department of Pediatrics, the First Hospital of Jilin University from September 2014 to September 2015 were recruited into this study. All patients met the following criteria: (1) children under 18 years, (2) confirmed diagnosis of IgAV according to the European League Against Rheumatism/ Pediatric Rheumatology International Trials Organization/ Pediatric Rheumatology European Society (EULAR/PRINTO/PRES) criteria [24], and (3) patients without other autoimmune diseases. The detailed EULAR/PRINTO/PRES diagnostic criteria are as follows: the presence of palpable purpura (mandatory criterion), together with at least one of following findings: (1) diffuse abdominal pain (abdominal involvement); (2) histopathology characterized by typical leukocytoclastic vasculitis (LCV) with predominant IgA deposits or proliferative glomerulonephritis with predominant IgA deposits; (3) acute arthritis or arthralgia (joint involvement); (4) renal involvement manifested by proteinuria (>0.3 g/24 h or >30 mmol/mg of urine albumin/creatinine ratio from the first morning urine sample), and/or hematuria (red blood cell [RBC] casts with >5 red blood cells/high-power field or ≥2+ on dipstick or presence of RBC casts in urinary sediment). According to presenting symptoms, the 27 patients in this study were further divided into five groups: skin type (*n* = 8), abdominal type (*n* = 8), kidney type (*n* = 5), joint type (*n* = 3) and the mixed type (patients presenting two or more non-purpura symptoms, *n* = 3).

Due to the self-limited and benign course of IgAV, symptom-oriented and supportive therapies were administered to patients following admission. For patients that presented severe gastrointestinal complications or proliferative glomerulonephritis, GC (intravenous methylprednisolone treatment starting at 3–5 mg/kg body weight/day, followed by tapering dosages until the relief of symptoms) were administered. Following treatment, remission was defined as the satisfaction of the following two criteria: (1) after 5–7 days of treatment, all skin purpura became obviously shallow or completely subsided, and no new rash appeared; (2) children with intestinal wall edema, arthralgia, hematuria and/or proteinuria, and other related symptoms experienced a dramatic relief of symptoms.

As controls, 15 age- and gender-matched healthy individuals (healthy controls, HC) were recruited into this study. Upon recruitment, the following clinical parameters were measured on all participants: routine blood test, serum immunoglobulin and complement level (by a specific protein analyzer SIEMENS BN-II, Germany), serum C-reactive protein (CRP) (using the QuikRead go CRP kit, Orion Diagnostica, Finland), urinary protein (using a P800 Biochemical Analyzer, Roche, Germany), and urinary RBC and white blood cell (WBC) count (using a UF-1000 automatic urine sediment analyzer, Sysmex, Japan).

### Cell isolation

Fasting venous blood samples were collected from HC and IgAV patients upon admission, and after disease remission (for IgAV patients only), respectively. Peripheral blood mononuclear cells (PBMCs) were isolated from individual patients and HC by density-gradient centrifugation using Ficoll-Paque Plus (Amersham Biosciences, Little Chalfont, UK) at 800 × g for 30 min at 25 °C.

### Flow cytometry analysis

Freshly isolated PBMCs (4 × 10^6^/mL) were cultured in 10 % fetal calf serum RPMI-1640 (Hyclone, Logan, UT, USA) in U-bottom 24-well tissue culture plates (Costar, Lowell, MA, USA), stimulated with or without 50 ng/mL of phorbol myristate acetate (PMA) plus 2 μg/mL of ionomycin (Sigma, St. Louis, MO, USA) for one hour, followed by treatment with Brefeldin A (10 μg/mL, GolgiStop™; BD Biosciences, San Jose, CA, USA) for an additional five hours. Then, these cells were stained in duplicate with BV510-anti-CD3, APC-H7-anti-CD4, BB515-anti-CXCR5, PE-Cy5-anti-CD45RA, PE-CF594-anti-CD279 and BV421-anti-CD278 (Beckton Dickinson, San Jose, CA, USA) at room temperature for 30 min. Subsequently, cells were fixed, permeabilized, and stained with PE-anti-IL-21 (Beckton Dickinson). The frequencies of distinct Tfh cells were analyzed by multicolor flow cytometry (FACSAria™ II, BD Biosciences), and data were processed using FlowJo software (v5.7.2; FlowJo, Ashland, OR, USA).

### Measurement of plasma IL-21 by cytometric bead array (CBA)

Plasma IL-21 concentrations were determined by a CBA human soluble protein master buffer kit (BD Biosciences) according to the manufacturer’s instructions, analyzed using a flow cytometer (FACSAria™ II, BD Biosciences), and quantified using the CellQuest Pro and CBA software (Becton Dickinson).

### Statistical analysis

Overall variations among the different groups were analyzed by one-way ANOVA. All data were presented as median and range. Student’s unpaired or paired *t*-test was appropriately chosen between groups. Mann–Whitney test was performed for nonparametric data between the two studied groups. The relationship between variables was analyzed by Pearson rank correlation test. All statistical analyses were performed using SPSS version 19.0 software. A two-tailed *P* value <0.05 was considered statistically significant.

## Results

### Clinical characteristics of children with IgAV

The general demographic and clinical characteristics of all participants are summarized in Table 1. According to the presenting symptoms, eight patients (29.63 %) presented with skin purpura (skin type), eight (29.63 %) with gastrointestinal tract discomfort (abdominal type), five (18.52 %) with microhematuria and/or mild proteinuria (1+ to 2+) (kidney type), three (11.1 %) with arthralgia and/or arthritis (joint type), and three (11.11 %) with two or more non-purpura symptoms (mixed type). Preceding upper airway infections were recorded in 20 (74.07 %) patients, and 23 (85.19 %) patients were tested positive for mycoplasma infection. Upon recruitment, the WBC count (*P* < 0.0001), platelet (*P* = 0.0045), serum IgA (*P* = 0.0097), IgE (*P* = 0.0371) and complement C4 (*P* = 0.0476) levels were significantly higher in IgAV patients than in HC (Table 1).

**Table 1:** The demographic and clinical characteristics of participants

	IgAV (*n =* 27)	Healthy Controls (*n =* 15)
Age, year	7 (3–13)	6 (2–14)
Female/Male	14/13	7/8
WBC, 10^9^/L	9.32 (4.23–19.33)*	7.5 (5.31–9.28)
Lymphocytes, 10^6^/L	3.96 (1.1–5.54)	3.57 (1.46–4.07)
Platelet, g/L	303 (188–463)*	298 (172–404)
Serum IgA, g/L	2.14 (0.95–5.91)*	1.47 (0.91–4.03)
Serum IgG, g/L	10.5 (0.95–17.4)	9.28 (1.03–15.22)
Serum IgM, g/L	1.18 (0.7–3.22)	1.07 (0.65–3.51)
Serum IgE, g/L	54.6 (16.7–657)*	22.1 (17.1–77.4)
Serum C3, g/L	1.29 (0.89–1.63)	1.35 (0.91–1.68)
Serum C4, g/L	0.34 (0.18–0.45)*	0.23 (0.16–0.41)
Serum CRP (mg/L)	7.23 (1.16–72.54)*	3.5 (0.82–5.1)

*P < 0.05, vs HC the values before treatment

### Detection of circulating Tfh cells

**Fig. 1:**
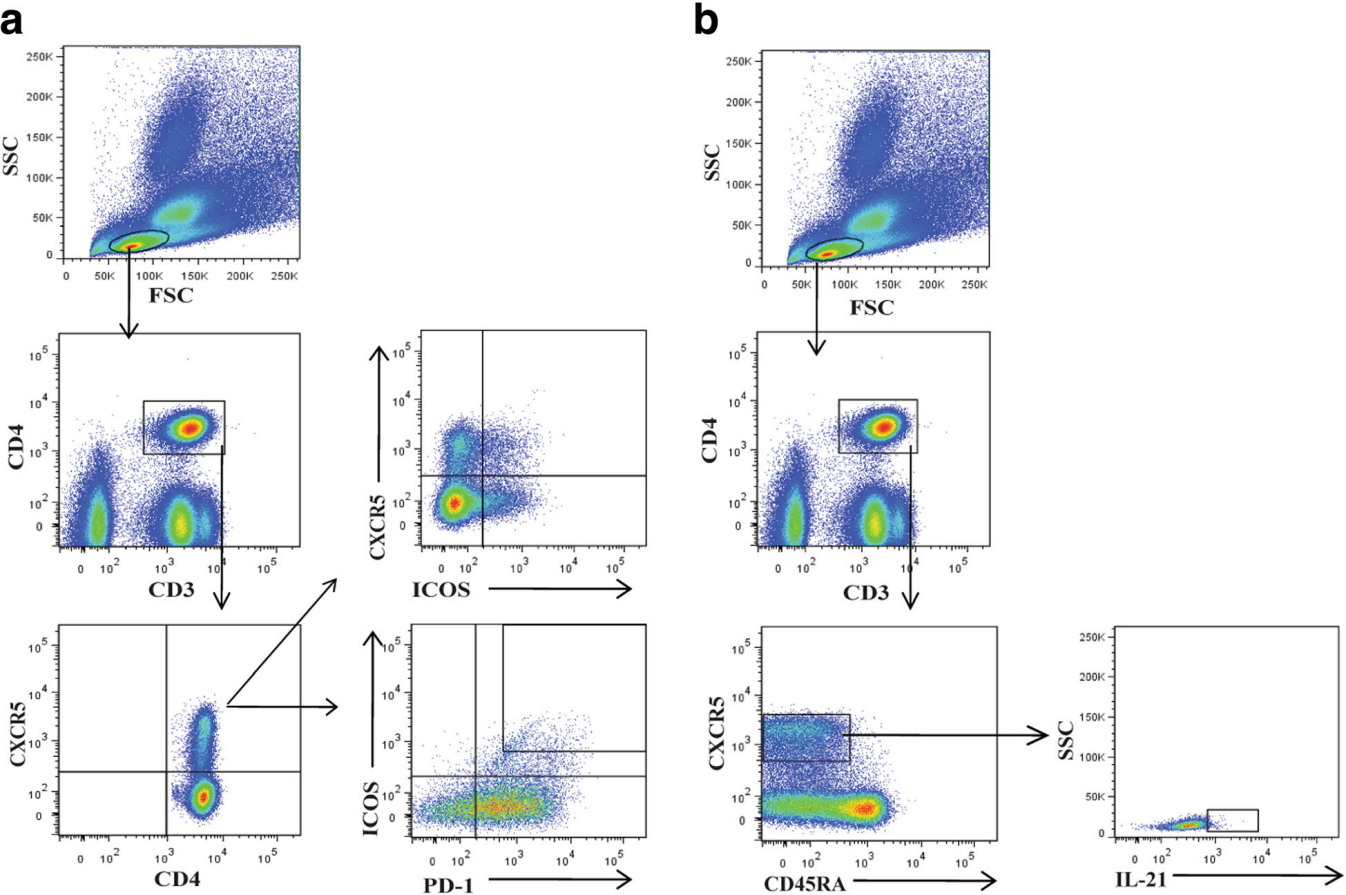
Detection of circulating Tfh cells by flow cytometry. PBMCs were isolated from IgAV patients (*n* = 27) and age- and gender-matched healthy controls (HC; *n* = 15), stained with fluorophore-conjugated antibody targeting indicated proteins, and analyzed by flow cytometry. **a** The gating strategy to identify CD4^+^CXCR5^+^, CD4^+^CXCR5^+^ICOS^+^, CD4^+^CXCR5^+^ICOS^+^PD-1^+^, CD4^+^CXCR5^+^ICOS^high^PD-1^high^, and CD4^+^CXCR5^+^ICOS^−^PD-1^+^ Tfh cells. **b** The gating strategy to identify CXCR5 + CD45RA-IL-21+ Tfh cells

In order to assess the significance of circulating Tfh cells in IgAV, focus was given on the following Tfh cells: CD4^+^CXCR5^+^, CD4^+^CXCR5^+^ICOS^+^, CD4^+^CXCR5^+^ICOS^+^PD-1^+^, CD4^+^CXCR5^high^ICOS^+^PD-1^high^, CD4^+^CXCR5^+^ICOS^−^PD-1^+^, and CXCR5^+^CD45RA^−^IL-21^+^, which were identified by flow cytometry (Fig. 1). CD4^+^CXCR5^+^ Tfh cells and its four subpopulations, CD4^+^CXCR5^+^ICOS^+^, CD4^+^CXCR5^+^ICOS^+^PD-1^+^, CD4^+^CXCR5^+^ICOS^high^PD-1^high^ and CD4^+^CXCR5^+^ICOS^−^PD-1^+^, were gated from CD3^+^CD4^+^ T cells (Fig. 1a); while CXCR5^+^CD45RA^−^IL-21^+^ Tfh cells were independently gated from CD3^+^CD4^+^ T cells (Fig. 1b).

### Association of different phenotypic subpopulations of Tfh cells and cytokine with IgAV symptoms and treatment options

Next, we analyzed the status of different subpopulations of Tfh cells and plasma Tfh cytokine IL-21 in IgAV, as well as their associations to IgAV symptoms and treatments.

**Fig. 2:**
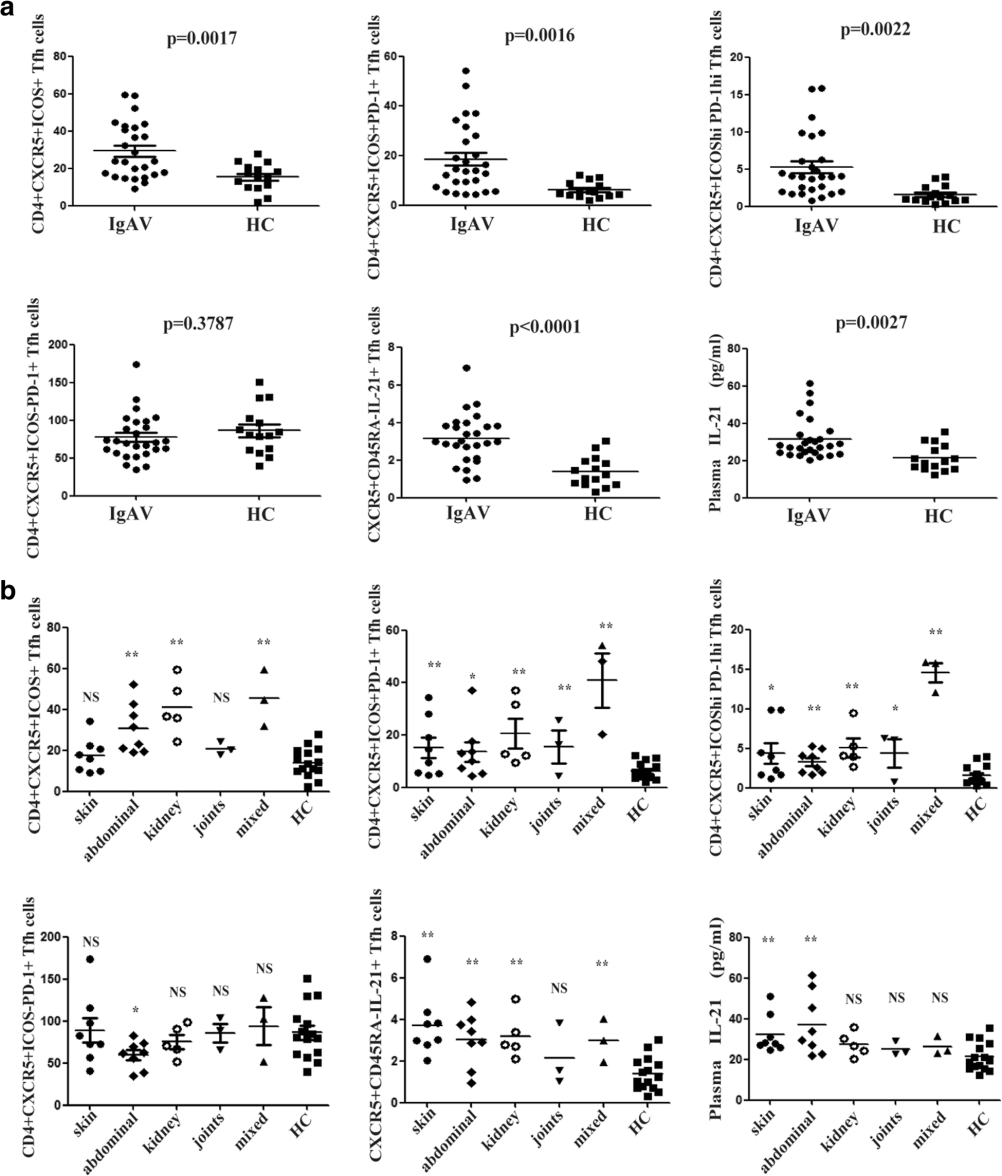
Association of different phenotypes of Tfh cells with IgAV symptoms and treatment options. The comparison of indicated Tfh cells and plasma IL-21 levels between IgAV patients and HC (**a**), between IgAV patients with different symptom types and HC (**b**). *P < 0.05, **P < 0.01, compared with the HC group; NS, not significant, when compared with the HC group

The frequencies of circulating CD4^+^CXCR5^+^ (data were not shown), CD4^+^CXCR5^+^ICOS^+^, CD4^+^CXCR5^+^ICOS^+^PD-1^+^, CD4^+^CXCR5^high^ICOS^+^PD-1^high^ and CXCR5^+^CD45RA^−^IL-21^+^ Tfh cells, as well as plasma IL-21 levels, were all significantly higher in IgAV patients than in HC (*P* < 0.05; Fig. 2); while the frequency of circulating CD4^+^CXCR5^+^ICOS^−^PD-1^+^ was not significantly different between these two groups (*P* > 0.05; Fig. 2).

Further analysis on the association of circulating Tfh cells or plasma IL-21 levels with the presenting symptoms of IgAV revealed different patterns of association (Fig. 2b): compared to levels in HC, CD4^+^CXCR5^+^ Tfh cells were significantly higher in patients with skin, kidney, joint and mixed types (*P* = 0.0069, 0.0233, 0.0236 and 0.0494, respectively), but not in those with abdominal type (*P* > 0.05, data were not shown); CD4^+^CXCR5^+^ICOS^+^ Tfh cells increased in patients with abdominal, kidney and mixed types (*P* = 0.0060, 0.0007 and 0.0005, respectively), but not in those with skin or joint type (*P* > 0.05); both CD4^+^CXCR5^+^ICOS^+^PD-1^+^ and CD4^+^CXCR5^+^ICOS^high^PD-1^high^ Tfh cells were dramatically elevated in patients with all symptom types; CXCR5^+^CD45RA^−^IL-21^+^ Tfh cells were significantly higher in patients with skin, abdominal, kidney and mixed types (*P* < 0.001, 0.0011, 0.0008 and 0.0081, respectively), but not in those with joint type (*P* > 0.05). Interestingly, CD4^+^CXCR5^+^ICOS^−^PD-1^+^ Tfh cells was significantly lower in patients with an abdominal type (*P* = 0.0412), but not in those with other types (*P* > 0.05), compared with HC. Furthermore, plasma IL-21 levels were significantly higher in patients with skin and abdominal types (*P* = 0.0052 and 0.0027, respectively), but not in those with kidney, joint or mixed type (*P* > 0.05), compared with HC.

When the association of different Tfh cells with treatment options (non-GC *vs.* GC) were analyzed among patients entering disease remission, no significant difference was detected in any of the Tfh cells or plasma IL-21 (*P* > 0.05, data were not shown).

### Alterations of Tfh cells and plasma IL-21 following treatment

**Fig. 3:**
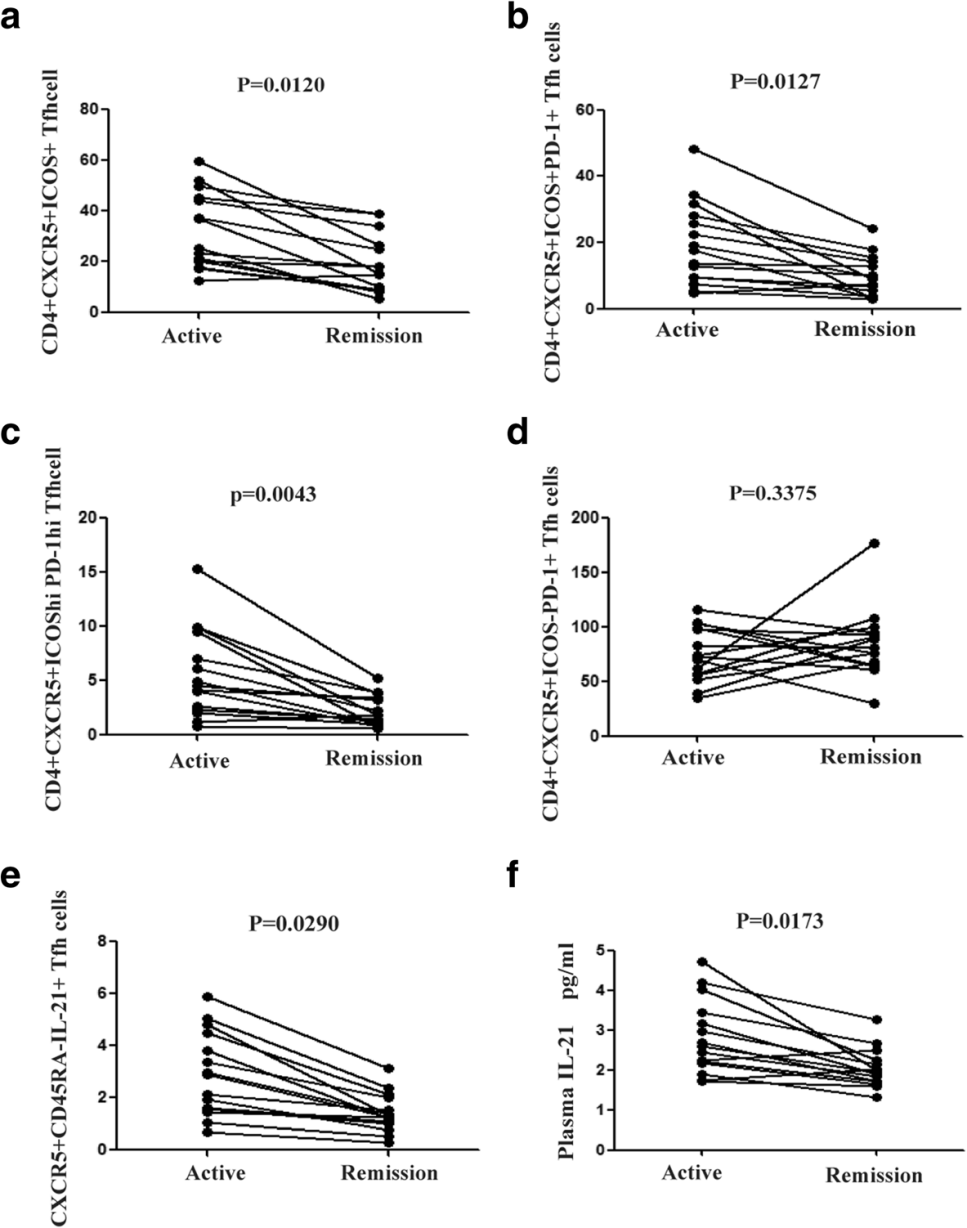
Treatment-induced alterations of different subpopulations of Tfh cells and plasma IL-21. After proper treatment, disease remission was achieved in 15 patients. The frequency of the indicated Tfh cells and plasma IL-21 levels were compared between the active and remission stages of the disease

Following admission, all patients received symptom-oriented and supportive therapies; and 25 patients achieved disease remission. Among these patients, 15 patients were examined for these subpopulations of Tfh cells before treatment during the active stage of the disease, as well as after treatment during the remission stage (Fig. 3). With disease remission, the frequencies of circulating CD4^+^CXCR5^+^ICOS^+^, CD4^+^CXCR5^+^ICOS^+^PD-1^+^, CD4^+^CXCR5^+^ICOS^high^PD-1^high^ and CXCR5^+^CD45RA^−^IL-21^+^ Tfh cells were significantly reduced from the corresponding value in the active stage (*P* = 0.0120, 0.0127, 0.0043 and 0.0290, respectively). No significant difference was detected in CD4^+^CXCR5^+^ICOS^−^PD-1^+^ cells following disease remission (*P* = 0.3375, Fig. 3). Meanwhile, plasma IL-21 levels also significantly decreased in the remission stage, when compared to the active stage (*P* = 0.0173, Fig. 3).

### Correlation between Tfh cells and serum IgA, C4 and plasma IL-21

**Fig. 4:**
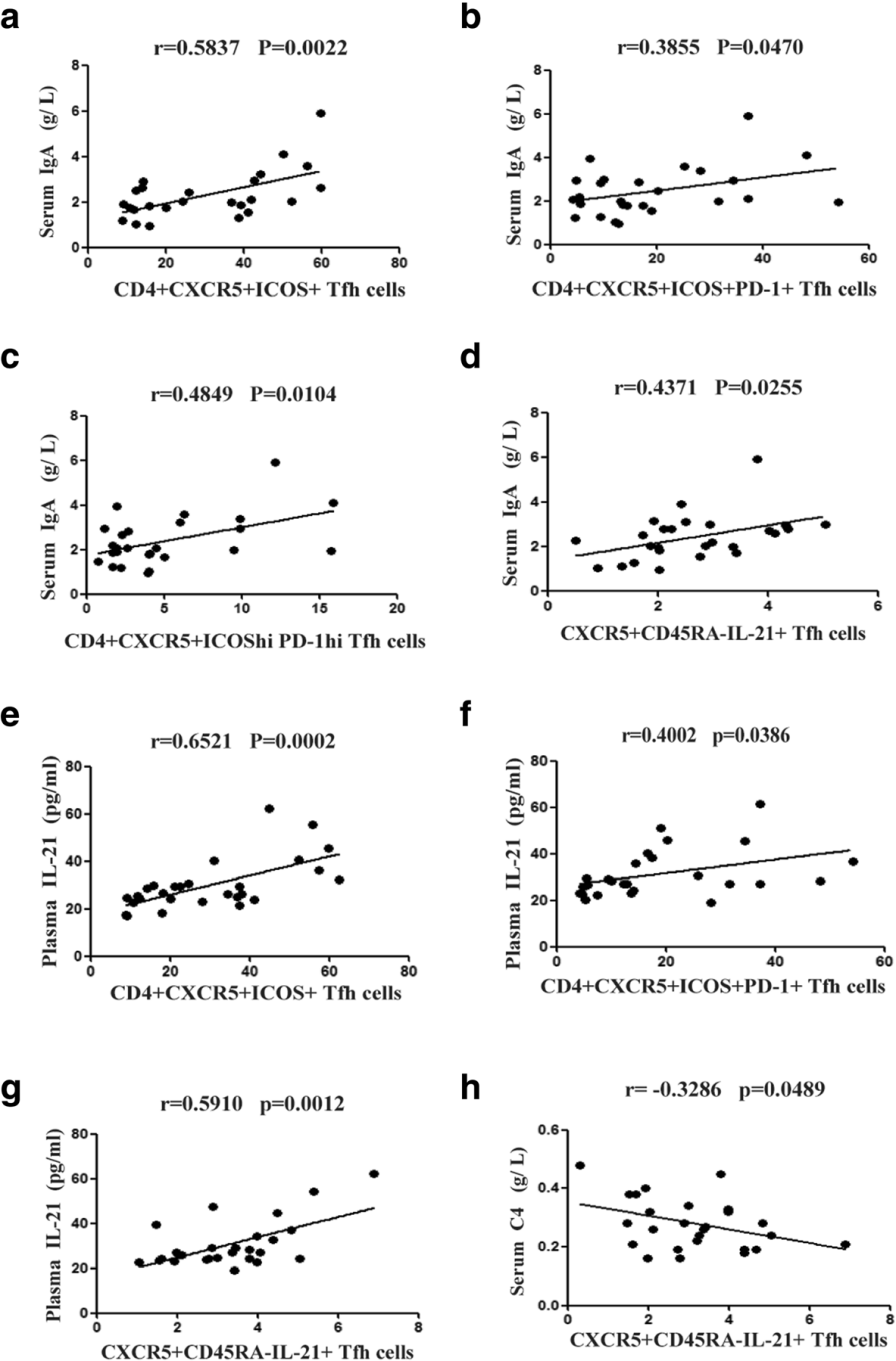
Correlation between different phenotypic subpopulations of Tfh cells and serum IgA, complement C4 or plasma IL-21. The correlation between the indicated Tfh cells and serum IgA (**a**-**d**), plasma IL-21 (**e**-**g**) and C4 (**h**) was analyzed by Pearson rank correlation test

When the correlation between different Tfh cells and different clinical parameters of IgAV were analyzed, it was found that circulating CXCR5^+^CD45RA^−^IL-21^+^ (*r* = 0.4371, *P* = 0.0255), CD4^+^CXCR5^+^ICOS^+^ Tfh cells (*r* = 0.5837, *P* = 0.0022), CD4^+^CXCR5^+^ICOS^+^PD-1^+^ (*r* = 0.3855, *P* = 0.0470) and CD4^+^CXCR5^+^ICOS^high^PD-1^high^ (*r* = 0.4849, *P* = 0.0104), but not CD4^+^CXCR5^+^ICOS^−^PD-1^+^ (*r* = −0.1618, *P* = 0.4201, data were not shown) Tfh cells, were significantly and positively correlated with serum IgA levels (Fig. 4a-d). Circulating levels of CD4^+^CXCR5^+^ICOS^+^ (*r* = 0.6521, *P* = 0.0002), CD4^+^CXCR5^+^ICOS^+^PD-1^+^ (*r* = 0.4002, *P* = 0.0386) and CXCR5^+^CD45RA^−^IL-21^+^ (*r* = 0.5910, *P* = 0.0012) Tfh cells were also significantly and positively correlated with plasma IL-21 levels (Fig. 4e-g). Furthermore, circulating CXCR5^+^CD45RA^−^IL-21^+^ Tfh cells (*r* = −0.3286, *P* = 0.0489) were the only cells significantly and negatively correlated with serum C4 levels (Fig. 4h).

## Discussion

In this study, we presented prime evidence that circulating CD4^+^CXCR5^+^ Tfh cells are not homogenous, but rather a heterogeneous population of cells distinguishable by combinations of Tfh phenotypic markers. Functionally, these phenotypic subpopulations are differentially regulated in IgAV patients presenting different patterns of association with the dominant symptoms of the disease, and un-equivalently correlated with key clinical IgAV parameters. Upon disease remission following treatment, these cells also responded differently. This is the first study that revealed the differential contributions of Tfh cells in IgAV pathogenesis and their alterations following disease progression.

Consistent with their specialized functions to help B cells in antibody production, the aberrant expansion of Tfh cells have been identified in autoimmune diseases including systemic lupus erythematosus (SLE), Sjogren’s syndrome and juvenile dermatomyositis; which are all characterized by the production of pathogenic autoantibodies [25, 26]. Among the plethora of autoimmune diseases, IgAV is a common connective tissue disease associated with the vascular deposition of IgA-dominant immunoglobulin complexes [1]. Most recently, scientists began to investigate the potential involvement of Tfh cells in IgAV, and two studies have both identified the expansion of circulating CD4^+^CXCR5^+^ICOS^+^ Tfh cells in IgAV patients, compared with cells in healthy controls [22, 23]. However, neither these two nor other studies explored the potential phenotypic subpopulations of Tfh cells in IgAV or their association with different clinical features of IgAV. Consistent with these two studies, we have shown that the frequency of CD4^+^CXCR5^+^ICOS^+^ Tfh cells in peripheral blood from IgAV patients were significantly higher than in healthy individuals. In addition, we have revealed that the expansion was not unique to CD4^+^CXCR5^+^ICOS^+^ cells, since the frequencies of circulating CD4^+^CXCR5^+^, CD4^+^CXCR5^+^ICOS^+^PD-1^+^, CD4^+^CXCR5^+^ICOS^high^PD-1^high^ and CXCR5^+^CD45RA^−^IL-21^+^ Tfh cells were also significantly higher in IgAV patients. Furthermore, correlation studies revealed that the frequencies of circulating CD4^+^CXCR5^+^ICOS^+^, CD4^+^CXCR5^+^ICOS^+^PD-1^+^, CD4^+^CXCR5^+^ICOS^high^PD-1^high^ and CXCR5^+^CD45RA^−^IL-21^+^ Tfh cells were significantly and positively correlated with serum IgA levels. In contrast, the frequency of CD4^+^CXCR5^+^ICOS^−^PD-1^+^ Tfh cells did not present any significant change in IgAV patients, compared to healthy individuals, which is consistent with the findings of Xie et al. [22]. Besides, CD4^+^CXCR5^+^ICOS^−^PD-1^+^ Tfh cells were not correlated with serum IgA levels. These data suggest that more than one phenotypic subpopulations of Tfh cells, but not all, may contribute to the pathogenesis and progression of IgAV.

Although not unique for Tfh cells, common Tfh cell markers are closely associated with the functions of these cells: chemokine receptor CXCR5 is important for B-cell homing to B cell follicles [27, 28]; PD-1 supports the survival and selection of high-affinity plasma cells in the germinal center [29]; ICOS is essential for the maintenance and function of Tfh in the germinal center [30, 31]; IL-21 critically regulates the growth, differentiation and class-switching of B cells [32, 33]. In circulating Tfh cells, the exact functions of each marker remains to be addressed; but they may not significantly differ from those in germinal center Tfh cells. All markers were identified in CD4^+^CXCR5^+^ Tfh cells. However, it is not known whether different combinations of these markers would generate distinct subpopulations of Tfh cells; and more importantly, whether these subpopulations would be functionally different from each other. In this study, we revealed that there are at least four different phenotypic subpopulations of circulating Tfh cells: CD4^+^CXCR5^+^ICOS^+^, CD4^+^CXCR5^+^ICOS^+^PD-1^+^, CD4^+^CXCR5^+^ICOS^high^PD-1^high^, CD4^+^CXCR5^+^ICOS^−^PD-1^+^ and CXCR5^+^CD45RA^−^IL-21^+^. Even though they may not be completely exclusive from each other, they did present varied biological functions in IgAV. CD4^+^CXCR5^+^ICOS^+^, CD4^+^CXCR5^+^ICOS^+^PD-1^+^, CD4^+^CXCR5^+^ICOS^high^PD-1^high^ and CXCR5^+^CD45RA^−^IL-21^+^ Tfh cells were all significantly expanded in the circulation of IgAV; and their levels lowered dramatically following effective treatment and disease remission. However, their frequencies varied with the dominant clinical symptoms presented in patients: CD4^+^CXCR5^+^ICOS^+^ cells were not significantly altered in patients with predominant skin or joint symptoms; CXCR5^+^CD45RA^−^IL-21^+^ cells were not significantly altered in patients with predominant joint symptoms; both circulating CD4^+^CXCR5^+^ICOS^+^PD-1^+^ and CD4^+^CXCR5^+^ICOS^high^PD-1^high^ were significantly expanded in IgA patients presenting with all dominant symptoms, in which CD4^+^CXCR5^+^ICOS^+^PD-1^+^ and CD4^+^CXCR5^+^ICOS^high^PD-1^high^ were most robustly expanded in patients with mixed symptoms, as well as in CXCR5^+^CD45RA^−^IL-21^+^ cells in those with skin symptoms. These findings imply that different subpopulations of Tfh cells differentially regulate the development of organ-specific symptoms.

Interestingly, although the frequency of circulating CD4^+^CXCR5^+^ICOS^−^PD-1^+^ Tfh cells did not change dramatically in IgAV patients compared to healthy individuals, their level was significantly lower in patients presenting abdominal symptoms. PD-1 and its ligands play a critical role in maintaining peripheral tolerance [34]. Signaling through PD-1 attenuates the signaling downstream of T cell receptors (TCR) and inhibits T cell expansion, cytokine production and cytolytic activity. In addition, PD-1 signaling inhibits the aberrant activation of T cells and benefits the induction of regulatory T cells [35–37]. The non-elevation of CD4^+^CXCR5^+^ICOS^−^PD-1^+^ Tfh cells in IgAV and its downregulation in patients presenting abdominal dominant symptoms may reflect a pathogenic mechanism during IgAV development, specifically the development of abdominal symptoms in IgAV; which would inhibit the potential immunosuppressive activity of Tfh cells, and thus shift the balance toward enhanced autoimmunity.

The characteristic cytokine produced by Tfh cells is IL-21, which is a type I cytokine with pleiotropic immune activities including regulating germinal center B-cell responses, isotype switching and the generation of memory B cells [38–40]. Both B and CD4^+^ T cells require IL-21 signaling for generating long-term humoral immunity [17, 41]. Many CD4^+^ T cells can produce IL-21, with the most abundant sources being Tfh and Th17 cells [42]. In this study, we found that plasma IL-21 levels were significantly elevated in IgAV patients, particularly in patients with dominant skin and abdominal symptoms; but not in patients with joint, kidney or mixed symptoms, as compared with healthy individuals. Following disease remission, elevated plasma IL-21 level was significantly reduced; suggesting that circulating IL-21 levels are sensitive indicators for active IgAV. Furthermore, we identified significant and positive correlations between plasma IL-21 levels with the frequency of circulating CXCR5^+^CD45RA^−^IL-21^+^, CD4^+^CXCR5^+^ICOS^+^PD-1^+^ and CD4^+^CXCR5^+^ICOS^+^ Tfh cells, implying that these three phenotypes of Tfh cells may contribute to IgAV development through the secretion of IL-21.

GC is a powerful anti-inflammatory drug, but we only use it for patients with severe symptoms. It can inhibit inflammation by downregulating T and B cell function and reducing cytokine production. In this study, we divided patients during the remission stage (*N* = 15) into the GC group (*n* = 5) and the non-GC group (*n* = 10). Surprisingly, the difference in Tfh cell subsets between these two groups was not significant. These findings also supported the notion that IgAV is a common kind of self-limiting disease, and its recovery is mainly based on its own re-established immune homeostasis. Although GC can rapidly relieve severe active symptoms, a small dose of GC does not lead to immunosuppression, Cushing’s syndrome, and other adverse reactions. In addition, this result does not reflect the value of GC on immune regulation due to the lack of long-term follow-up and tracing studies; hence, we could not absolutely determine the value of GC therapy for IgAV, especially for the long-term prognosis of renal type. The number of patients in this study is few, and there is a need to explore more patients, especially with different prognosis types, in future studies.

Although the major conclusions drawn from this study are limited by the relatively small sample size, these provide seminal findings that would guide future, larger-scale studies. Furthermore, it is important to further characterize the Tfh cell subsets from this study, both on phenotypes and functions; and compare them with Tfh cell subsets defined from other studies such as Th1, Th2 and Th17 subsets [20].

In summary, this study identified multiple phenotypic subpopulations of Tfh cells, namely, CD4^+^CXCR5^+^ICOS^+^, CD4^+^CXCR5^+^ICOS^+^PD-1^+^, CD4^+^CXCR5^+^ICOS^high^PD-1^high^, CD4^+^CXCR5^+^ICOS^−^PD-1^+^ and CXCR5^+^CD45RA^−^IL-21^+^; which are functionally important for the pathogenesis of IgAV. The levels of these subpopulations in the peripheral circulation of IgAV in patients were significantly higher than in healthy individuals; they also correlate with IgAV clinical markers including circulating IL-21 and IgA levels, which decreased following disease remission. In addition, these subsets presented differential associations with the organ-specific symptoms of IgAV. Therefore, these Tfh cells not only serve as indicators of IgAV symptoms and progression, but also becomes therapeutic targets that enable the individualized or symptom-oriented treatment of IgAV.

## Conclusion

IgAV is the most common cutaneous vasculitis in children, immune system disorders play a key role in its pathogenesis. Here, we found Tfh cells may differentially contribute to the development of IgAV or predict disease progression. These findings provide novel insights in the pathogenesis of IgAV, and this may be new targets for intervention of organ-specific IgAV.

## Abbreviations

CBA: Cytometric bead array
HC: Healthy controls
IgAV: IgA vasculitis
LCV: Leukocytoclastic vasculitis
PBMC: Peripheral blood mononuclear cells
Tfh cells: Follicular helper T cells
